# Screening and the analysis of genotypic and phenotypic characterization of glucose-6-phosphate dehydrogenase (G6PD) deficiency in Fujian province, China

**DOI:** 10.3389/fgene.2024.1422214

**Published:** 2024-07-15

**Authors:** Jinfu Zhou, Yinglin Zeng, Jianping Tang, Shihong Chen, Guilin Li, Xiaolong Qiu, Peiran Zhao, Ting Huang, Jinying Luo, Na Lin, Liangpu Xu

**Affiliations:** ^1^ Medical Genetic Diagnosis and Therapy Center, Fujian Key Laboratory for Prenatal Diagnosis and Birth Defect, Fujian Maternity and Child Hospital College of Clinical Medicine for Obstetrics and Gynecology and Pediatrics, Fujian Medical University, Fuzhou, Fujian Province, China; ^2^ School of Medical Tcehnology and Engineering, School of Public Health, Fujian Medical University, Fuzhou, Fujian Province, China; ^3^ Department of Preventive Medicine, School of Public Health, Fujian Medical University, Fuzhou, Fujian Province, China; ^4^ Obstetrics and Gynecology Department, Fujian Maternity and Child Hospital College of Clinical Medicine for Obstetrics and Gynecology and Pediatrics, Fujian Medical University, Fuzhou, Fujian Province, China

**Keywords:** G6PD deficiency, incidence rate, genotype, phenotype, southeastern China

## Abstract

**Introduction:**

Glucose-6-phosphate dehydrogenase (G6PD) deficiency is a common X-linked hereditary disorder in southern China. However, the incidence rate of G6PD deficiency and the frequency of the most common *G6PD* gene variants vary widely. The purpose of this study was to investigate the prevalence, genotype, and phenotypic features of G6PD deficiency in neonates in Fujian province, southeastern China.

**Methods:**

This retrospective cohort study enrolled 2,789,002 newborns (1,521,431 males and 1,267,571 females) based on the newborn screening program for G6PD deficiency in Fujian Province between January 2010 and December 2021.

**Results:**

Of the 2,789,002 newborns enrolled, 26,437 cases were diagnosed (22,939 males and 3,498 females), and the estimated prevalence of G6PD deficiency in Fujian province was 0.95%. The prevalence was significantly higher among males (1.51%) than in females (0.28%) (*p* < 0.00001). Among the 3,198 patients with G6PD deficiency, 3,092 cases (2,145 males and 947 females) were detected to have G6PD gene variants. The top six prevalent genotypes identified represented 90.84% (2095/3,198) of the total and included c.1376G > T (44.93%), c.1388G > A (18.42%), c.1024C > T (9.32%), c.95A > G (8.69%), c.392G > T (5.25%), and c.871G > A (4.22%). The frequency of genotypes with c.1388G > A, c.1024C > T, and c.871G > A was higher in males in the Fujian province than in females, while the frequency of genotypes with c.1376G > T was lower. Furthermore, when comparing the enzyme activities of the top six prevalent genotypes, there were significant differences in the enzyme activities among the genotypes of male hemizygotes and female heterozygotes. According to the new classification of G6PD variants proposed by the World Health Organization (WHO), the variants with c.1376G > T, c.95A > G, and c.871G > A were recognized as Class A, while the c.392G > T, c.1388G > A, and c.1024C > T were recognized as Class B.

**Discussion:**

To the best of our knowledge, this study is the first to systematically describe the overview of epidemiological characteristics of newborn G6PD deficiency in Fujian province, China, including the screening rate, incidence rate, and variant spectrum. Additionally, we elucidated the relationship between the distribution of enzyme activity with specific mutations and their WHO classification patterns. Our results could provide strategies for screening, diagnosis, and genetic counseling of G6PD deficiency in this area.

## 1 Introduction

The most prevalent and the first-to-be recognized red blood cell enzyme deficiency, glucose-6-phosphate dehydrogenase (G6PD) deficiency, is caused by hereditary mutations in the X-linked gene, *G6PD*. G6PD is a housekeeping enzyme and essential for shielding cells from oxidative damage. The clinical presentations of G6PD deficiency are highly variable, including acute hemolytic anemia (AHA), chronic nonspherocytic hemolytic anemia (CNSHA), neonatal jaundice and asymptomatic conditions (Luzzatto et al., 2020). AHA in individuals with G6PD deficiency is a medical emergency and even life-threatening, which is mainly triggered by the ingestion of fava beans, by several drugs (e.g., primaquine, rasburicase), or rarely by infection (Luzzatto and Arese, 2018; La Vieille et al., 2019; Garcia et al., 2021; Ruan et al., 2022). Hence, early diagnosis is crucial for preventing the ingestion of fava beans and drug-induced AHA in China. Currently, approximately over 500 million people worldwide are affected by G6PD deficiency. Nonetheless, there are significant regional and ethnic variations in the prevalence of G6PD deficiency, ranging from 0% in the indigenous American inhabitants to more than 20% in some areas of Africa and Asia (Arain and Bhutani, 2014; Galatas et al., 2017; Nuinoon et al., 2022). Epidemiologically, G6PD deficiency is more prevalent in the populations of southern China, particularly in the Guangdong (Liang et al., 2022), Guangxi (Yan et al., 2006), Hainan (Zeng et al., 2022), and Jiangxi (Liu et al., 2020) provinces.

The *G6PD* gene is located in Xq28 and contains 13 exons, encoding a 515-amino acids long polypeptide chain. The form of dimer and tetramer of G6PD are enzymatically active, while the monomer has no enzyme activity. More than 270 variants of the *G6PD* gene have been identified worldwide, with the vast majority being missense mutations and a very few being small in-frame deletions (Luzzatto et al., 2020). Most mutations of the *G6PD* gene lead to impaired folding or impaired stability of the dimer or tetramer of G6PD, resulting in decreased stability (Cunningham et al., 2017; Praoparotai et al., 2020; Geck et al., 2023). However, the variants spectrum of the *G6PD* gene varies from country to region (Saad et al., 1997; Karimi et al., 2008; Leslie et al., 2013; Carter et al., 2018; Sathupak et al., 2021). More than 35 pathogenic variants have been found in the Chinese population. Among these variants, c.1376 G > T, c.1388 G > A, c.95A > G, c.1024C > T, and c.871 G > A are the top five *G6PD* gene variants frequencies (Liu et al., 2020). Moreover, 1376G > T, 1388G > A, and 95A > G are only found in the Chinese ancestry. However, the frequency of the most common *G6PD* gene variants varies in different provinces, with regional and ethnic characteristics (Liu et al., 2020). Furthermore, investigating the correlations between genotype and phenotype plays a significant role in genetic counseling and management of G6PD deficiency. Although there are currently few clinical studies (Wang et al., 2021; Zhang et al., 2024), further exploration of the relationship between *G6PD* gene mutations and G6PD enzyme activity requires a large-scale G6PD deficiency cohort.

The classification of G6PD-deficient variants was first proposed in 1966 and amended in 1985 by the World Health Organization (WHO), including Class I, <10% residual enzymatic activity with CNSHA, Class II, <10% residual enzymatic activity without CNSHA, Class III, 10%–60% residual enzymatic activity, Class Ⅳ, 60%–150% residual enzymatic activity, and Class Ⅴ, more than twice normal enzymatic activity, which is still in use today. Currently, the adjusted male median (AMM) is defined as the 100% enzyme activity that is widely used (Ley et al., 2020; Pfeffer et al., 2020; Xuan-Rong Koh et al., 2023; Võ et al., 2024). In 2022, the WHO proposed a new classification of *G6PD* variants in homozygous and hemizygous individuals, including four classes: A, B, C, and U (https://www.who.int/publications/m/item/WHO-UCN-GMP-MPAG-2022.01).

At the beginning of 2010, the newborn screening (NBS) program for G6PD deficiency was gradually conducted in Fujian province. Although a few studies on G6PD deficiency were conducted in some regions of Fujian Province (Chen et al., 2018; Wang et al., 2021), the prevalence and genotypes of G6PD deficiency in Fujian Province have not been elucidated. Therefore, this study aimed to investigate the prevalence and genotype features of G6PD deficiency in the Fujian neonatal cohort born between 2010 and 2021. Furthermore, we also investigated the relationship between *G6PD* gene mutations and G6PD enzyme activity. The results of this study could elucidate the epidemiological characteristics and provide an accurate prevention and management strategy for G6PD deficiency in the Fujian province.

## 2 Methods

### 2.1 Study population

This retrospective cohort study enrolled 2,789,002 newborns (1,521,431 males and 1,267,571 females) based on NBS for G6PD deficiency in the Fujian Province between January 2010 and December 2021. This study was performed in accordance with the guidelines of the Declaration of Helsinki and approved by the Ethics Review Committee of Fujian Provincial Maternity and Child Hospital (approval number: 2023KYLLR01003). All participants’ parents or guardians decided and signed the informed consent after providing a fully description of the study’s objectives.

### 2.2 NBS for G6PD deficiency

The workflow of NBS was based on a previously described procedure (Qiu et al., 2023; Zhou et al., 2023). Briefly, After drying at room temperature, the dried blood spot samples were transported to the NBS center the next day using a cold-chain transportation system. The G6PD activity was detected by a G6PD assay kit (Labsystems, Finland) using the fluorescence method, strictly according to the manufacturer’s instructions. The fluorescence signal was measured using the time-resolved fluoroimmunoassay analyzer, 1420 VICTOR™ D (Wallac Oy, Finland). A threshold value of G6PD < 2.6 U/g Hb for males and <3.3 U/g Hb for females was determined as positive for G6PD deficiency screening.

### 2.3 Diagnosis for G6PD deficiency

Participants who tested positive for NBS were recalled and further detected using a nitroblue tetrazolium (NBT) G6PD/6PGD (6-phosphogluconate dehydrogenase) test for diagnosis. The NBT G6PD/6PGD quantification ratio assay kit (Fenghua Co., Ltd, Guangzhou, China) was used for assaying G6PD activity, following the manufacturer’s protocols. G6PD/6PGD ratio <1.0 was diagnosed as G6PD deficiency.

### 2.4 Genotype analysis

Nucleic acid was extracted from dried blood spot specimens using the Lab-aid 824 nucleic acid extractor (Xiamen Zeesan Biotech Co., Ltd, China), strictly according to the manufacturer’s protocol. The *G6PD* gene mutations test kit (Xiamen Zeesan Biotech Co., Ltd, China) was utilized to identify 12 of the most prevalent variations of the *G6PD* gene in the Chinese population using fluorescence melting curve analysis. The detectable variants included c.95A > G, c.383T > C, c. 392G > T,c.487G > A, c.517T > C, c.592C > T, c.871G > A, c.1004C > A, c.1024C > T, c.1360C > T, c.1376G > A, and c.1388G > A. Both PCR amplification and result analysis were carried out using the SLAN-96S Real-Time PCR System (Xiamen Zeesan Biotech Co., Ltd, China). The interpretation of specimen genotyping was based on the Human Gene Mutation Database (HGMD-referencing the GRCh37/hg19 human gene database).

### 2.5 Statistical analyses

SPSS 18.0 was used to analyze all the data. The prevalence of G6PD deficiency was presented as a percentage (%), and the categorical data were expressed as proportions. Measurement data were presented as mean ± standard deviation. A chi-square test or one-way analysis of variance was performed to analyze the comparison between subgroups. Statistical significance was set at *p* < 0.05.

## 3 Results

### 3.1 NBS and diagnosis for G6PD deficiency

**FIGURE 1:**
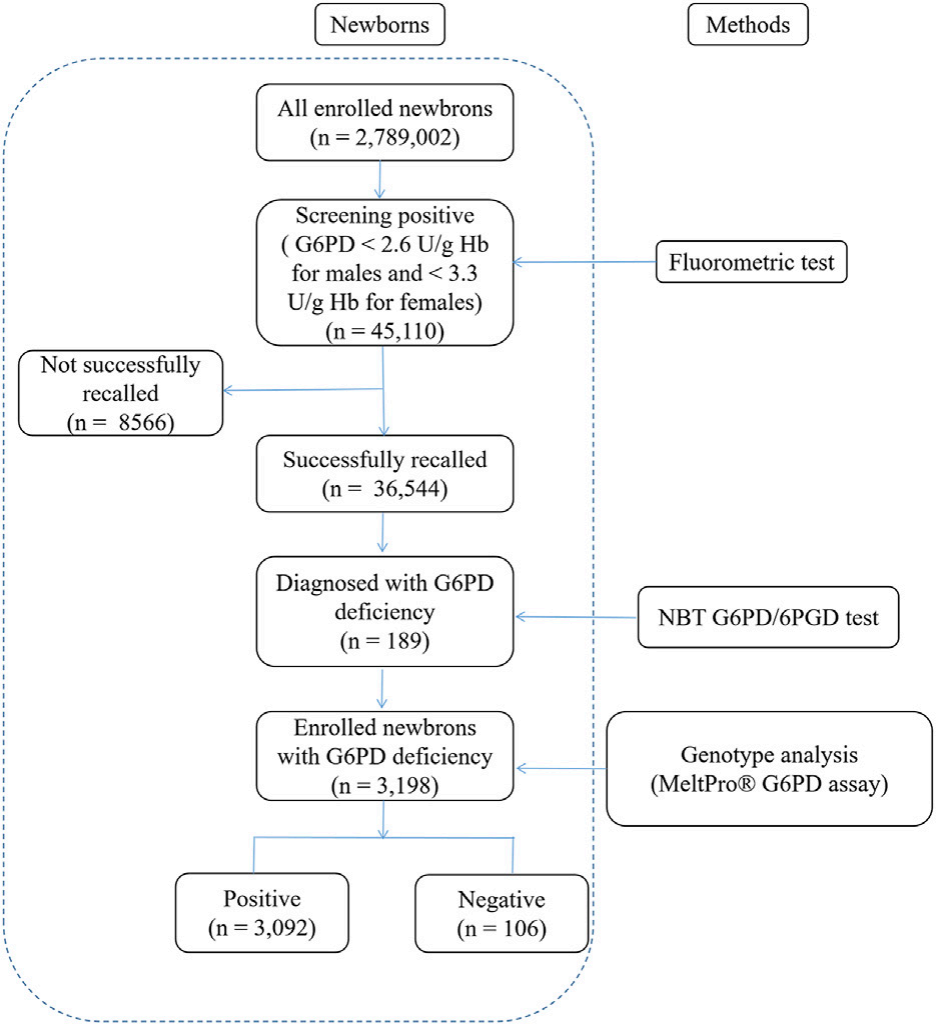
Flowchart showing an overview of the results from newborn screening and diagnostic tests for G6PD deficiency. G6PD, Glucose-6-phosphate dehydrogenase.

Over 12 years, 2,789,002 newborns (1,521,431 males and 1,267,571 females) in the Fujian province underwent NBS for G6PD deficiency. A total of 45,110 cases were found to be positive upon screening, yielding a positivity rate of 1.67%. A total of 36,544 cases were recalled for further diagnosis, with a recall rate of 81.01%. Of these, 26,437 cases were eventually diagnosed (22,939 males and 3,498 females) by the NBT G6PD/6PGD test, and the consistency rate between initial screening and diagnosis was 72.34% (Figure 1). Thus, the estimated prevalence of G6PD deficiency in Fujian province was 0.95%, and the prevalence was significantly higher among males (1.51%) than in females (0.28%) (χ2 = 11,173.8, *p* < 0.0001).

### 3.2 Genotype analysis of G6PD deficiency

To provide more insights into the mutation spectrum of the *G6PD* gene in the newborn population in Fujian Province, 3,198 affected newborns underwent mutation analysis for the 12 most frequent mutation loci in the Chinese population. Among the 3,198 patients with G6PD deficiency, 3,092 cases (2,145 males and 947 females) have detectable variants, while the other 106 patients (62 males and 44 females) did not have any identifiable variant. The frequency of various *G6PD* genotypes is shown in Table 1, including 2,145 males as hemizygotes, 932 females as heterozygotes, and 15 females as homozygotes. Among the 932 female heterozygotes, there were 893 single heterozygotes and 39 compound heterozygotes. The top six prevalent genotypes identified represented 90.84% (2,905/3,198) of the total and were c.1376G > T (44.93%), c.1388G > A (18.42%), c.1024C > T (9.32%), c.95A > G (8.69%), c.392G > T (5.25%), and c.871G > A (4.22%). A comparison of the relative frequency of the top six common genotypes identified in the G6PD deficiency newborns between males and females was then performed. The results showed that the frequency of genotypes with c.1388G > A, c.1024C > T, and c.871G > A was higher in males in Fujian province than in females, while the frequency of genotypes with c.1376G > T was lower (Table 2).

**TABLE 1:** The number and frequency of different G6PD genotypes in the Fujian province.

Mutation name	Genotypes	gDNA position (GRCh37)	Amino acid Substitution	Gene region	Male (2,207)	Female (991)		Total (3,198)	RF (%)
					Hemizygous mutation	Homozyous mutation	Heterozygous mutation		
Canton	c.1376G > T	chrX:153,760,484C > A	*p*.Arg459Leu	Exon 12	959	13	465	1,437	44.93
Kaiping	c.1388G > A	chrX:153,760,472C > T	*p*.Arg463His	Exon 12	453	1	135	589	18.42
Chinese-5	c.1024C > T	chrX:153,761,184G > A	*p*.Leu342Phe	Exon 9	231		67	298	9.32
Gaohe	c.95A > G	chrX:153,774,276T > C	*p*.His32Arg	Exon 2	184		94	278	8.69
Qing Yuan	c.392G > T	chrX:153,763,476C > A	*p*.Gly131Val	Exon 5	131		37	168	5.25
Viangchan, Jammu	c.871G > A	chrX:153,761,337C > T	*p*.Val291Met	Exon 9	86		49	135	4.22
Maewo, Chinese-2, Kalo	c.1360C > T	chrX:153,760,605G > A	*p*.Arg454Cys	Exon 11	48	1	24	73	2.28
Mahidol	c.487G > A	chrX:153,762,710C > T	*p*.Gly163Ser	Exon 6	33		10	43	1.34
Nankang	c.517T > C	chrX:153,762,680 A > G	*p*.Phe173Leu	Exon 6	12		5	17	0.53
Fushan	c.1004C > A	chrX:153,761,204G > T	*p*.Ala335Asp	Exon 9	6		1	7	0.22
Shunde	c.592C > T	chrX:153,762,605G > A	*p*.Arg198Cys	Exon 6	1		3	4	0.12
Salerno	c.383T > C	chrX:153,763,485A > G	*p*.Leu128Pro	Exon 6	1		3	4	0.12
	c.95A>G/c.1024C > T						1	1	0.03
	c.95A>G/c.1360C > T						1	1	0.03
	c.95A>G/c.1376G > T						8	8	0.25
	c.95A>G/c.1388G > A						3	3	0.09
	c.392G > T/c.1024C > T						1	1	0.03
	c.392G > T/c.1360C > T						1	1	0.03
	c.392G > T/c.1376G > T						2	2	0.06
	c.392G > T/c.1388G > A						1	1	0.03
	c.517T > C/c.1376G > T						1	1	0.03
	c.871G > A/c.1024C > T						1	1	0.03
	c.871G > A/c.1376G > T						1	1	0.03
	c.1024C > T/c.1360C > T						1	1	0.03
	c.1024C > T/c.1376G > T						6	6	0.19
	c.1024C > T/c.1388G > A						1	1	0.03
	c.1360C > T/c.1376G > T						3	3	0.09
	c.1360C > T/c.1388G > A						1	1	0.03
	c.1376G > T/c.1388G > A						6	6	0.19

Abbreviation: G6PD, glucose-6-phosphate dehydrogenase; RF, relative frequency.

**TABLE 2:** Comparisons of the relative frequency of the top six common genotypes identified in the G6PD deficiency newborns between male and female.

	c.1376G > T (n/N, %)	c.1388G > A (n/N, %)	c.1024C > T (n/N, %)	c.392G > T (n/N, %)	c.871G > A (n/N, %)	c.1360C > T (n/N, %)
Male	959/2,207, 43.45	453/2,207, 20.52	231/2,207, 10.47	184/2,207, 8.34	131/2,207, 5.94	86/2,207, 3.90
Female	478/991, 48.23	136/991, 13.72	67/991, 6.76	94/991, 9.48	37/991, 3.73	49/991, 4.94
T or χ^2^	6.319	21.06	11.12	1.136	6.663	1.857
*P*	0.012	0.000	0.001	0.286	0.01	0.173

Abbreviation: G6PD, glucose-6-phosphate dehydrogenase.

### 3.3 Correlation of *G6PD* genotypes and enzyme activity

**FIGURE 2:**
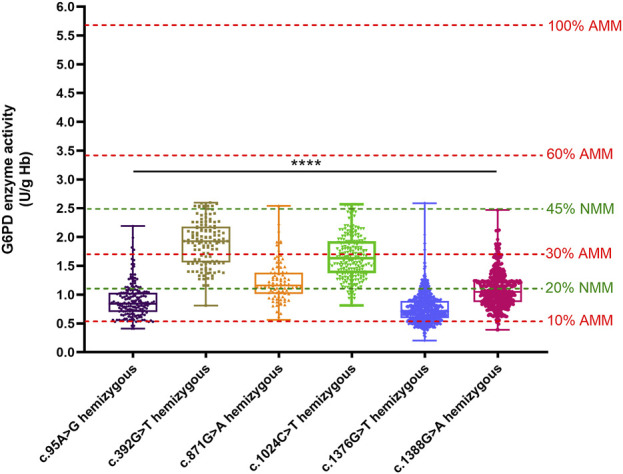
Box plot of the enzyme activity of females heterozygous for each mutation and males hemizygous for each mutation. The red four reference lines in the panel for male hemizygotes indicate 10%, 30%, 60% and 100% of the AMM, respectively. The two green lines represent the reference lines for Classes A and B of the new WHO classification. The enzyme activity between any two genotypes of male hemizygotes genotypes had a significant difference (****p < 0.0001).

**FIGURE 3:**
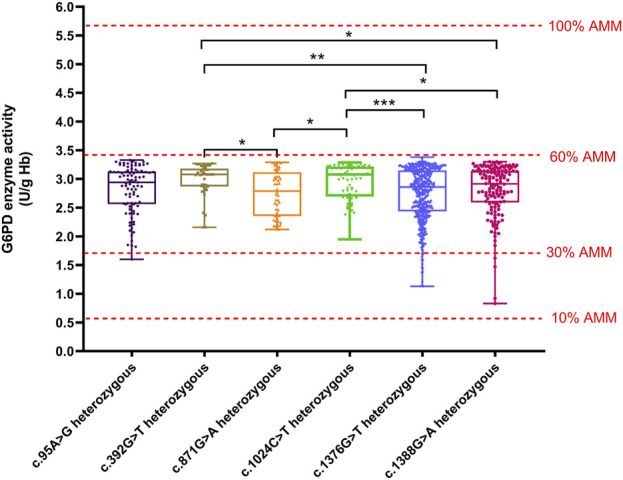
Box plot of the enzyme activity of females heterozygous for each mutation. The red four reference lines in the panel for male hemizygotes indicate 10%, 30%, 60% and 100% of the AMM, respectively. Among the genotypes of female heterozygotes, the enzyme activity of G6PD deficiency newborns carrying c.392G>T and c.1024C>T higher than that in c.871G>A, c.1376G>T, and c.1388G>A (*p < 0.05, **p < 0.01, and ***p < 0.001).

We compared the enzyme activities between G6PD-deficient newborns carrying the top six common genotypes. The results indicated there were significant differences in the enzyme activities among the genotypes of male hemizygotes and female heterozygotes (*p* < 0.01), as shown in Table 3. Among the genotypes of male hemizygotes, the c.392G > T genotype had the highest enzyme activity, while the c.1376G > T genotype had the lowest enzyme activity. Moreover, the enzyme activity between any two genotypes of male hemizygotes genotypes had a significant difference (*p* < 0.001), as shown in Figure 2. Moreover, among the genotypes of female heterozygotes, the enzyme activity of G6PD-deficient newborns carrying c.392G > T and c.1024C > T was higher than that in those carrying c.871G > A, c.1376G > T (44.93%), and c.1388G > A (Figure 3).

**TABLE 3:** Comparisons of the G6PD enzyme activity of the top six common genotypes identified in the G6PD deficiency newborns in male and female.

Genotype	Male hemizygous		Female heterozygous	
	Number of carriers	G6PD activity (U/g Hb)	Number of carriers	G6PD activity (U/g Hb)
c.95A > G	184	0.89 ± 0.29	94	2.81 ± 0.43
c.392G > T	131	1.90 ± 0.39	37	2.98 ± 0.29
c.871G > A	86	1.23 ± 0.36	49	2.75 ± 0.40
c.1024C > T	231	1.66 ± 0.39	67	2.94 ± 0.34
c.1376G > T	959	0.76 ± 0.25	465	2.74 ± 0.47
c.1388G > A	453	1.10 ± 0.31	135	2.79 ± 0.45
*F* value	582.3		4.132	
*p*-value	0.000		0.001	

**FIGURE 4:**
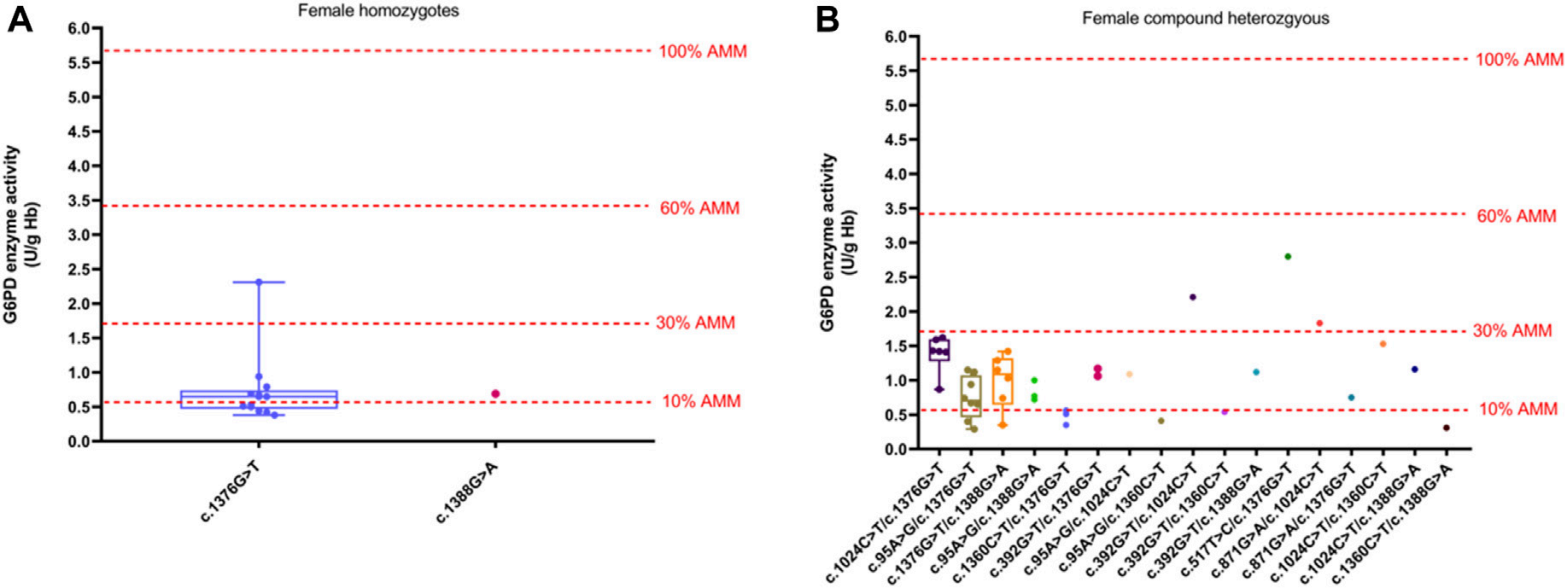
Distribution of the G6PD activities of different variants. **(A)** G6PD activities for female homozygotes. **(B)** G6PD activities for female compound heterozygotes. The red four reference lines in the panel for male hemizygotes indicate 10%, 30%, 60% and 100% of the AMM, respectively.

The method of calculation of AMM value was based on previous studies (Xuan-Rong Koh et al., 2023). Therefore, the AMM value was 5.69 U/g Hb in this study. According to the WHO classification, the variant with the residual enzyme activity was <0.569 U/g Hb (10% of AMM value) without CNSHA or 0.569–3.414 U/g Hb (10%–60% of AMM value), classified into Class II or Class III in this study, respectively. The enzyme activity values of the top six common genotypes were less than 60% of the AMM in both hemizygous males and heterozygous females. Moreover, the enzyme activity values ranged from 10% to 60% of the AMM in heterozygous females (Figure 3). The enzyme activity values of the variants with c.871G > A, c.392G > T, and c.1024C > T were between 10% and 60% of the AMM in hemizygous males (Figure 1). We also calculated the 30% AMM value (1.707 U/g Hb), which is as an acceptable level of G6PD activity for primaquine administration (Domingo et al., 2013). In addition, distribution of the G6PD activities for female homozygotes and compound heterozygotes was showned in Figure 4.

The percent activity value from the genotypically normal male median (NMM) value is recommended according to the WHO classification of the variants of the *G6PD* gene in 2022. Accordingly, the calculated NMM value in this study was 5.54 U/g Hb. Thus, the variant with the median of G6PD activity <1.11 U/g Hb (<20% of the NMM) was classified as WHO Class A, and the median of G6PD activity was <2.49 U/g Hb (<45% of the NMM) was classified as WHO Class B. In this study, we found the enzyme activity values of the variants with c.1376G > T and c.95A > G were almost lower than 20% of the NMM in hemizygous males, while the c.392G > T and c.1024C > T were between 20% and 45% of the NMM (Figure 2).

## 4 Discussion

Up to date, China has been certifed malaria free by the WHO on 30 June 2021. Therefore, the use of antimalarial drugs can lead to severe hemolysis in individuals with G6PD deficiency, which is rare in China. Thus, the use of antimalarial drugs cause severe haemolysis in G6PD-defcient individuals. However, the ingestion of fava beans, some oxidizing drugs (e.g., aspirin, sulfadiazine) and traditional Chinese medicines (e.g., berberine, honeysuckle) cancause a risk of haemolysis in G6PD defcient individuals in China (Lin et al., 1998; Chen et al., 2021; Zhang et al., 2022). Terefore, it is also essential to test for G6PD deficiency. NBS for G6PD deficiency was firstly conducted in the South region of China (Jiang et al., 2014; Yang et al., 2015). Later, it was expanded to other regions of China for its clinical significance in the identification of neonatal jaundice and acute or chronic hemolysis (Mei et al., 2022; Gao et al., 2023; Li et al., 2024).

To the best of our knowledge, this study includes the largest scale to provide an overview of epidemiological characteristics of G6PD deficiency in Fujian Province, including prevalence, common genotype frequency, and the relationship between *G6PD* genotypes and enzyme activity. The prevalence of G6PD deficiency is 4.95% worldwide (Nkhoma et al., 2009; Luzzatto et al., 2020). In Arab Countries, the incidence rate ranges from 2% to 31% (Alangari et al., 2023), while the prevalence of G6PD deficiency varies from 3.8% to 15% in South Asian countries (Arain and Bhutani, 2014). The prevalence of G6PD was 0.67%–0.77% in Chinese newborns, and the prevalence noted in southern China (0.95%) was significantly higher than that in northern China (0.028%), especially the incidence rates in Guangdong, Hainan, Jiangxi, and Guangxi exceeding 2% (Liu et al., 2020). The result of our study showed that the estimated prevalence rate of G6PD deficiency in the area was 0.95%, which was the same as the average incidence rate in southern China. Nevertheless, the incidence rate in this area is higher than that of several southern areas in China, such as Shanghai (0.112%), Zhejiang (0.251%), Yunnan (0.302%), and Sichuan (0.313%), which fully reflects the regional differences in the prevalence of G6PD deficiency. Moreover, the ratio of male-to-female with G6PD deficiency was 6.46:1, and the prevalence was significantly higher among males than in females, which is in line with the hallmark of an X-linked incomplete dominant inheritance pattern. It is noted that the quantitative G6PD activity test using fluorescence methods can accurately identify hemizygous males, compound heterozygous females, and homozygous females. However, G6PD heterozygous females cannot be accurately recognized using these quantitative biochemical methods, which will lead to the heterozygous females cannot been detected. In addition, due to the fact that hemolysis in patients with thalassemia can stimulate the body to produce a large number of red blood cells, some researchers have found that the G6PD activity in patients with thalassemia is higher than that in healthy individuals (Warny et al., 2015; Luzzatto et al., 2020). Talassemia has been reported to be relatively highly prevalent in Fujian province (Huang et al., 2019; Chen et al., 2022), which may lead to missed diagnosis of G6PD deficiency.

In addition to being used for the diagnosis of G6PD deficiency, genotyping of *G6PD* is also mainly used for epidemiological investigations and further exploration research on genotype-phenotype correlations. Except for a few rare small deletions, the *G6PD* gene mutations are almost all single nucleotide substitutions in the Chinese population (Lin et al., 2018; Shen et al., 2022). Although the prevalence of *G6PD* mutations varies genetically among different regions and races, the 12 *G6PD* variants included in this study for genotyping of *G6PD* are the most prevalent in China.

In this study, 44 genotypes were identified in 3,092 cases, accounting for 96.69% of the total cases of G6PD deficiency. Notably, 17 compound heterozygous genotypes and three types of homozygous genotypes were found in females. The most common genotypes in the Fujian province were c.1376G > T (44.93%), c.1388G > A (18.79%), and c.1024C > T (9.32%), which accounted for 73.04% of the total G6PD deficiency. These results were comparable to those in other Chinese provinces, such as Guangdong (Lin et al., 2018), Hubei (72.23%) (Shen et al., 2022), Guangxi (75.3%) (Yan et al., 2006), and Taiwan (70.9%) (Chiang et al., 1999). However, the highest frequency of the genotypes in Hubei province and Guangxi province is c.1388G > A, while the highest frequency in Guangdong and Taiwan is c.1376G > T, which is consistent with this region. The top six prevalent genotypes identified represented 90.84% (2,905/3,198) of the total in the Fujian province, which is equivalent to the proportions in the provinces of Guangxi, Guangdong, and Hainan (Liu et al., 2020). For the newborns with G6PD deficiency but no identifiable variants, in addition to using second-generation sequencing methods for detection in the future studies, we will also strengthen follow-up work on clinical manifestations and related biochemical testing to determine whether there is existence of an acquired form of G6PD deficiency (Pes and Dore, 2022). Furthermore, the frequency of genotypes with c.1388G > A, c.1024C > T, and c.871G > A was found to be higher in male hemizygotes in the Fujian province than those in female heterozygotes, while the frequency of genotypes with c.1376G > T was lower in male hemizygotes. Further research is needed to determine whether this is related to geography or sex differences.

It is important to clarify the relationship between specific genotypes and phenotypes based on a relatively large sample size in this study. As expected for an X-linked inheritable disease, among the top six most common mutations in this study, the G6PD activities were significantly lower in hemizygous males than those in heterozygous females. Differences in G6PD activity were observed among the top six common variants in male or female neonates, which is consistent with the results of previous studies (He et al., 2020; Li et al., 2023) However, some studies have found that there are no differences in G6PD activity among the genotypes in heterozygous females (Wang et al., 2021; Zhang et al., 2024), which may be related to the relatively small sample size.

To further understand the correlation between genotype and phenotype, most variants have been divided into five classes based on their enzymatic activity and clinical outcome by the WHO in the past few decades (Garcia et al., 2021). Most genetic variants identified in the Chinese population are grouped into Class II or III. Nevertheless, there is an inconsistency between the actual classification and the WHO class for specific variants in different studies (He et al., 2020; Wang et al., 2021). In this study, the enzyme activity values of the variants with c.392G > T and c.1024C > T were between 10% and 60% of the AMM in hemizygous males, which is consistent with previous reports (Minucci et al., 2012). According to the WHO class, c.95A > G and c.871G > A are identified as Class II. In addition, c.1376G > T and c.1388G > A are also identified as Class II in the previous studies (Calabrò et al., 1993; Wagner et al., 1996). However, most cases with c.1388G > A, c.95A > G, and c.871G > A in this study displayed residual enzymatic activities associated with class III. Additionally, 78.53% of cases with c.1376G > T were Class III.

Due to the considerable overlap between Class II and III variants, the WHO proposed a new classification system in 2022. The new classification system includes four classes: A (<20% of enzymatic activity/CNSHA), B (<45% of enzymatic activity/triggered AHA), C (60%–150% of enzymatic activity/no hemolysis), and U (any enzymatic activity/uncertain clinical significance). In the previous study, the variants with c.392G > T, c.1024C > T, c.1376G > T, c.1388G > A, c.95A > G, and c.871G > A were identified as Class A (Li et al., 2023). The enzyme activity values of the variants with c.1376G > T and c.95A > G were almost lower than 20% of the NMM in hemizygous males in this study, while the c.392G > T and c.1024C > T were between 20% and 45% of the NMM. Thus, the variants with c.1376G > T and c.95A > G were recognized as Class A, while the c.392G > T and c.1024C > T were recognized as Class B. The median G6PD activity in cases with variants c.1388G > A and c.871G > A was higher and lower than 20% of NMM, respectively. Therefore, the variants c.1388G > A and c.871G > A are identified as Class B and Class A.

To the best of our knowledge, this study is the first to systematically describe the overview of epidemiological characteristics of newborn G6PD deficiency in Fujian province, China, including the screening rate, incidence rate, and variant spectrum. Furthermore, we elucidated the relationship between the distribution of enzyme activity with specific mutations and their WHO classification patterns. In summary, early NBS and timely diagnosis for G6PD deficiency could provide strategies for genetic counseling and scientifically preventing the occurrence of serious complications, such as hemolysis.

## Data Availability

The original contributions presented in the study are included in the article/Supplementary Material, further inquiries can be directed to the corresponding authors.
